# Supplementary material for: General transcription factor from Escherichia coli with a distinct mechanism of action
Source: Nat Struct Mol Biol. 2024 Jan 4;31(1):141–9. doi: 10.1038/s41594-023-01154-w (PMC10803263; doi:10.1038/s41594-023-01154-w)
Supplement: Supplementary file 1 — Reporting Summary [file 41594_2023_1154_MOESM1_ESM.pdf]

nature portfolio

Corresponding author(s): *Double-anonymous peer review submissions: write DAPR and your manuscript number here instead of author names.*

Last updated by author(s): *YYYY-MM-DD*

Reporting Summary

Nature Portfolio wishes to improve the reproducibility of the work that we publish. This form provides structure for consistency and transparency in reporting. For further information on Nature Portfolio policies, see our Editorial Policies and the Editorial Policy Checklist.

Statistics

For all statistical analyses, confirm that the following items are present in the figure legend, table legend, main text, or Methods section.

n/a	Confirmed	
☐	☒	The exact sample size (*n*) for each experimental group/condition, given as a discrete number and unit of measurement
☐	☒	A statement on whether measurements were taken from distinct samples or whether the same sample was measured repeatedly
☐	☒	The statistical test(s) used AND whether they are one- or two-sided<br>*Only common tests should be described solely by name; describe more complex techniques in the Methods section.*
☐	☒	A description of all covariates tested
☐	☒	A description of any assumptions or corrections, such as tests of normality and adjustment for multiple comparisons
☐	☒	A full description of the statistical parameters including central tendency (e.g. means) or other basic estimates (e.g. regression coefficient) AND variation (e.g. standard deviation) or associated estimates of uncertainty (e.g. confidence intervals)
☐	☒	For null hypothesis testing, the test statistic (e.g. *F*, *t*, *r*) with confidence intervals, effect sizes, degrees of freedom and *P* value noted<br>*Give P values as exact values whenever suitable.*
☒	☐	For Bayesian analysis, information on the choice of priors and Markov chain Monte Carlo settings
☒	☐	For hierarchical and complex designs, identification of the appropriate level for tests and full reporting of outcomes
☒	☐	Estimates of effect sizes (e.g. Cohen's *d*, Pearson's *r*), indicating how they were calculated

Our web collection on statistics for biologists contains articles on many of the points above.

Software and code

Policy information about availability of computer code

Data collection	Mass spectrometry data were collected using Thermo Scientific Xcalibur v4.1.
Data analysis	Mass spectrometry data were analyzed using MaxQuant v2.0.1. Next-generation sequencing data were analyzed using Bowtie2 v2.4, Samtools v1.13 and custom R scripts. Statistical analysis was performed using R v4.1, including packages Rsubread, vsn, qvalue. Sequence alignment was done using MAFFT v7.487. Leginon v1.0 was used to collect electron microphotographs. Cryo-EM data were processed using CTFFIND4 v4.0.8, Gautomatch v0.56, RELION v3.1.2, cryoSPARC v3.2.0. Structure visualization was done in Chimera v1.16, map resolution was calculated by ResMap v1.1.4. Model was build in COOT v0.9.8 and refined in Phenix v1.18.2.

For manuscripts utilizing custom algorithms or software that are central to the research but not yet described in published literature, software must be made available to editors and reviewers. We strongly encourage code deposition in a community repository (e.g. GitHub). See the Nature Portfolio guidelines for submitting code & software for further information.

Data

Policy information about availability of data

All manuscripts must include a data availability statement. This statement should provide the following information, where applicable:

- Accession codes, unique identifiers, or web links for publicly available datasets
- A description of any restrictions on data availability
- For clinical datasets or third party data, please ensure that the statement adheres to our policy

Mass spectrometry data is available at PRIDE database with project ID PXD039446.
Next-generation sequencing data is available at SRA with BioProject accession number PRJNA924329.
Structure coordinates are available at PDB with accession code 8FTD and at EMDB with code EMD-29423.

Research involving human participants, their data, or biological material

Policy information about studies with human participants or human data. See also policy information about sex, gender (identity/presentation), and sexual orientation and race, ethnicity and racism.

Reporting on sex and gender	Not applicable
Reporting on race, ethnicity, or other socially relevant groupings	Not applicable
Population characteristics	Not applicable
Recruitment	Not applicable
Ethics oversight	Not applicable

Note that full information on the approval of the study protocol must also be provided in the manuscript.

Field-specific reporting

Please select the one below that is the best fit for your research. If you are not sure, read the appropriate sections before making your selection.

☒ Life sciences ☐ Behavioural & social sciences ☐ Ecological, evolutionary & environmental sciences

For a reference copy of the document with all sections, see nature.com/documents/nr-reporting-summary-flat.pdf

Life sciences study design

All studies must disclose on these points even when the disclosure is negative.

Sample size	Study was done with bacterial cultures that were used in their entirety for high-throughput experiments. Volume of cultures was chosen to provide enough material (protein, protein-bound DNA, or RNA) for analyses, based on published estimates of corresponding molecules copy number (for reference see https://doi.org/10.1038/nbt.3418, https://www.thermofisher.com/us/en/home/references/ambion-tech-support/rna-tools-and-calculators/macromolecular-components-of-e.html). For plating experiments, bacterial cultures were diluted so that individual colonies could be grown and counted.
Data exclusions	No data were excluded from analyses.
Replication	Experiments were performed in replicates on different days. Two replicates were done for ChIP-seq experiment, three replicates for RNA-seq and LC-MS experiments.
Randomization	Not applicable. This is not a confirmatory nor clinical study, and does not involve animals or humans.
Blinding	Not applicable. This is not a confirmatory nor clinical study, and does not involve animals or humans.

Reporting for specific materials, systems and methods

We require information from authors about some types of materials, experimental systems and methods used in many studies. Here, indicate whether each material, system or method listed is relevant to your study. If you are not sure if a list item applies to your research, read the appropriate section before selecting a response.

Materials & experimental systems

n/a	Involved in the study
☐	☒ Antibodies
☒	☐ Eukaryotic cell lines
☒	☐ Palaeontology and archaeology
☒	☐ Animals and other organisms
☒	☐ Clinical data
☒	☐ Dual use research of concern
☒	☐ Plants

Methods

n/a	Involved in the study
☐	☒ ChIP-seq
☒	☐ Flow cytometry
☒	☐ MRI-based neuroimaging

Antibodies

Antibodies used: Pierce anti-DYKDDDDK antibody covalently coupled to Magnetic Agarose (cat # A36797).

Validation: Per manufacturers (Thermo Scientific) manual, the product is high-affinity rat monoclonal antibody (clone L5) that is covalently attached to a magnetite-embedded agarose core particle with binding capacity of equal or more than 3.2 mg of DYKDDDDK-tGFP-His protein (32 kDa) per 1 ml of settled beads. Manufacturer demonstrated use of affinity resin for isolation of various N- and C-terminally FLAG-tagged proteins with high yield and purity.

Plants

Seed stocks: Not applicable

Novel plant genotypes: Not applicable

Authentication: Not applicable

ChIP-seq

Data deposition

☒ Confirm that both raw and final processed data have been deposited in a public database such as GEO.

☒ Confirm that you have deposited or provided access to graph files (e.g. BED files) for the called peaks.

Data access links
May remain private before publication.
https://www.ncbi.nlm.nih.gov/sra/?term=PRJNA924329

Files in database submission: ctl_1_R1.fastq.gz, ctl_1_R2.fastq.gz, ctl_2_R1.fastq.gz, ctl_2_R2.fastq.gz, ceda_1_R1.fastq.gz, ceda_1_R2.fastq.gz, rpoc_1_R1.fastq.gz, rpoc_1_R2.fastq.gz, rpoc_2_R1.fastq.gz, rpoc_2_R2.fastq.gz

Genome browser session (e.g. UCSC): Not applicable.

Methodology

Replicates: Two technical replicates for each bacterial strain were done on different days.

Sequencing depth:
75-nt paired reads.
experiment, total reads, uniquely mapped (-F 4 -q 30)
ctl_1, 32543296, 31289041
ceda_1, 25728884, 24639037
rpoc_1, 37064686, 21616956
ctl_2, 29238808, 27442010
ceda_2, 26947978, 25521810
rpoc_2, 38674750, 23064615

Antibodies: Pierce™ Anti-DYKDDDDK Magnetic Agarose, Thermo Scientific Cat A36797.

Peak calling parameters: 3 sigma above background level measured in control samples (IP from FLAG-less E. coli strain).

Data quality: Only properly mapped pairs of reads with mapping quality above 10 were considered for analysis.

Software: For reads mapping and filtering bowtie2 and samtools were used. Peak calling was done in R. R scripts are provided with submission.
