# Supplementary figures and images for: General transcription factor from Escherichia coli with a distinct mechanism of action
Source: Nat Struct Mol Biol. 2024 Jan 4;31(1):141–9. doi: 10.1038/s41594-023-01154-w (PMC10803263; doi:10.1038/s41594-023-01154-w)

Number of TSS

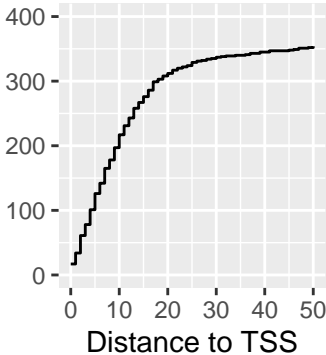

Supplement: Supplementary file 4 — Supplementary Code. [file 41594_2023_1154_MOESM4_ESM.zip › ceda/ceda-chip-seq/results/ceda_tss_dist.pdf]

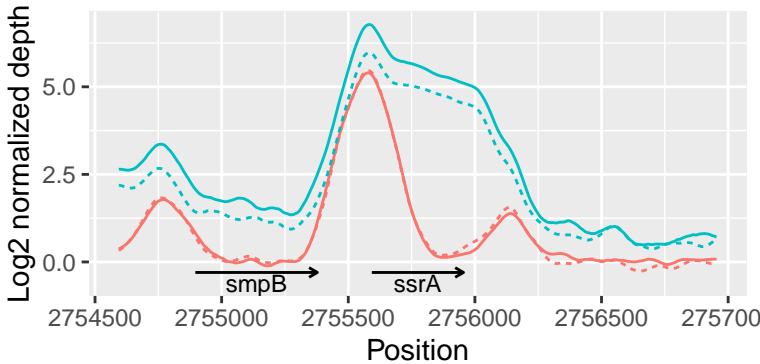

Supplement: Supplementary file 4 — Supplementary Code. [file 41594_2023_1154_MOESM4_ESM.zip › ceda/ceda-chip-seq/results/profile_ssra.pdf]

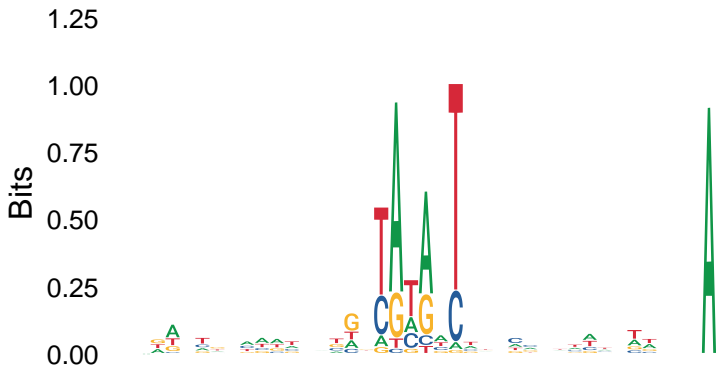

Supplement: Supplementary file 4 — Supplementary Code. [file 41594_2023_1154_MOESM4_ESM.zip › ceda/ceda-chip-seq/results/ceda_peaks_aln_logo.pdf]

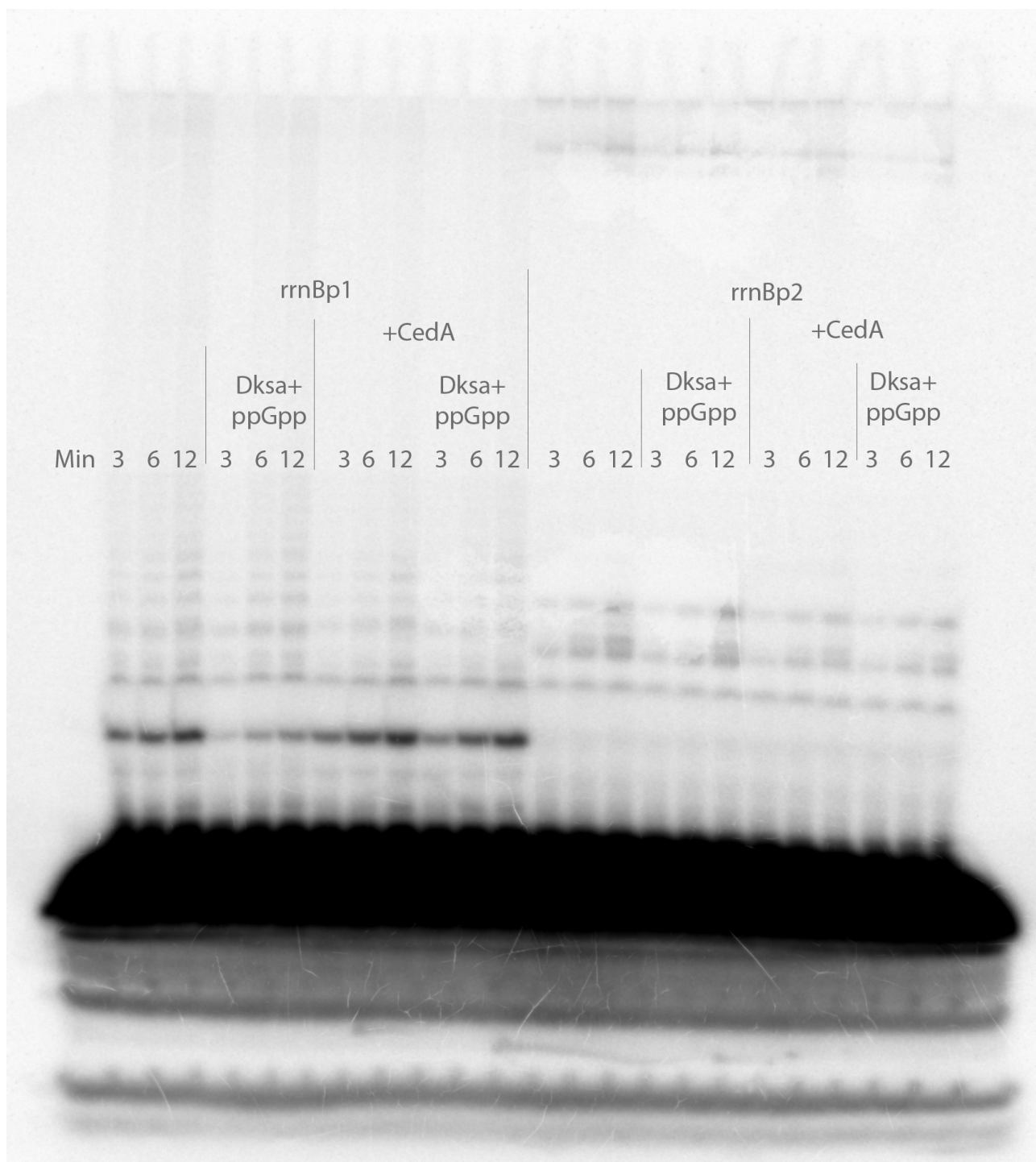

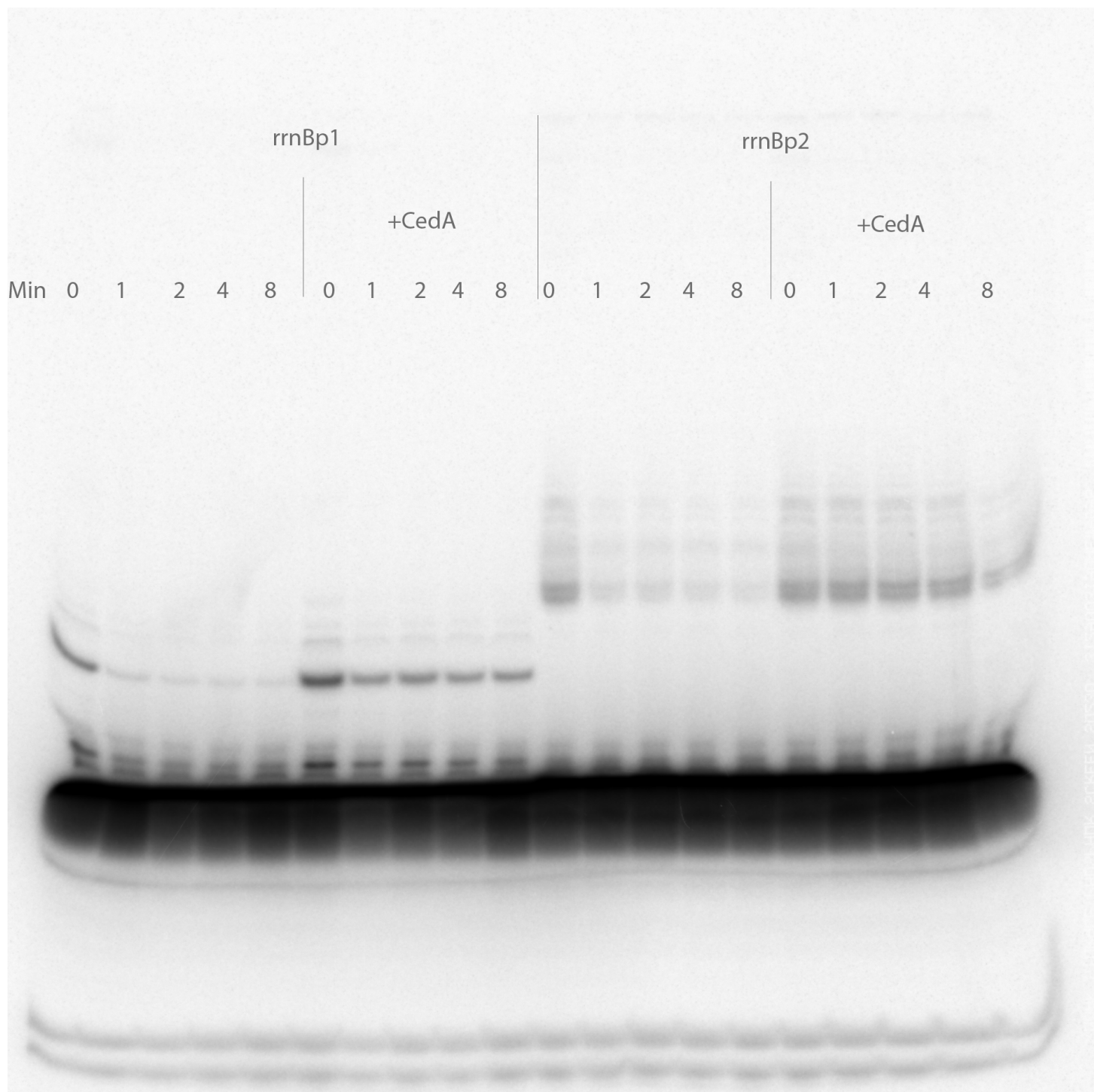

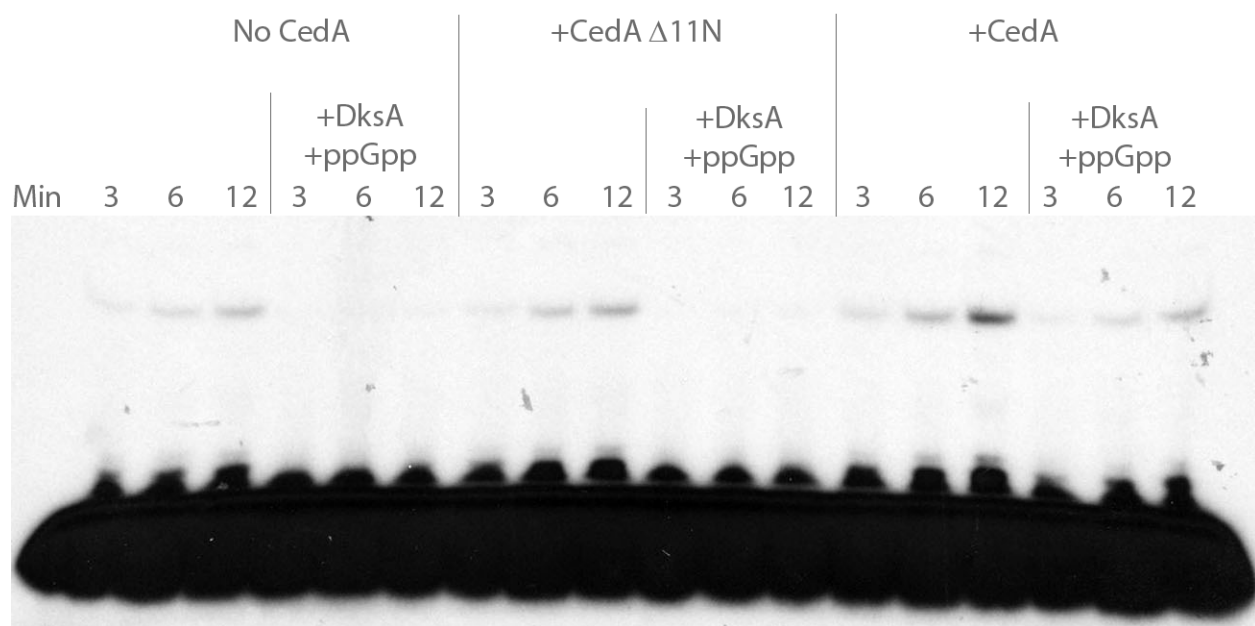

Supplement: Supplementary file 5 — Unprocessed autoradiographs and scans of X-ray film. [file 41594_2023_1154_MOESM5_ESM.pdf]

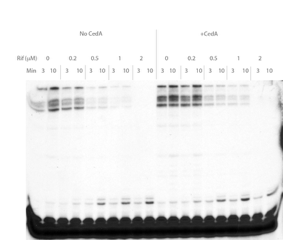

Supplement: Supplementary file 6 — Ct values obtained in RT–qPCR experiments for katE mRNA and fold changes of colony numbers in plating experiments. [file 41594_2023_1154_MOESM6_ESM.tiff]
